# International Regensburg Center for Interventional Immunology (RCI) symposium on “Synthetic immunology and environment-adapted redirection of T cells”, 17–18 July, 2019, Regensburg, Germany

**DOI:** 10.1007/s00262-019-02467-w

**Published:** 2020-03-02

**Authors:** Philipp Beckhove, Matthias Edinger, Markus Feuerer, Luca Gattinoni, Hinrich Abken

**Affiliations:** 1 Regensburg Center for Interventional Immunology (RCI) and Chair for Interventional Immunology, University Regensburg, Regensburg, Germany; 2 Regensburg Center for Interventional Immunology (RCI), José Carreras Center and Clinic III Internal Medicine, University Hospital Regensburg, Regensburg, Germany; 3 Regensburg Center for Interventional Immunology (RCI) and Chair for Immunology, University Regensburg, Regensburg, Germany; 4 Regensburg Center for Interventional Immunology (RCI) and Chair for Functional Immune Cell Modulation, University Regensburg, Regensburg, Germany; 5 Regensburg Center for Interventional Immunology (RCI) and Chair for Genetic Immunotherapy, University Regensburg, Franz-Josef-Strauss-Allee 11, 93053 Regensburg, Germany

**Keywords:** Synthetic immunology, Cell therapy, CAR, TCR, Treg cells, Regensburg center for interventional immunology (RCI)

## Introduction

Immunotherapeutic strategies are well established for the treatment of autoimmune diseases and in transplantation medicine since many years; only recently significant improvements in cancer therapy were achieved with the introduction of therapeutic antibodies along with conventional chemotherapy. A paradigm shift for the immunotherapeutic treatment of cancer began with the introduction of checkpoint inhibitors illustrating the potency of immunotherapeutic strategies by releasing a broad productive anti-tumor and eventually autoimmune response. The most promising, albeit also the most sophisticated and challenging approach of interventional immunology, is the targeted redirection of immune cells by genetic engineering as individually tailored “living drugs”, as illustrated by the success of chimeric antigen receptor (CAR)-modified T cells for the treatment of leukemia and lymphoma. Emerging synthetic immunology approaches provide a broad set of novel tools for immune cell (re-)programming to equip effector cells with defined, specific and enhanced anti-tumor capacities, probably also for solid tumors. Being dedicated to this fascinating field of research the Regensburg Center for Interventional Immunology (RCI) hosted the “Synthetic Immunology and Environment-adapted Redirection of T cells” symposium in the Thon-Dittmer-Palais Regensburg on 17–18 July, 2019. The aim of the symposium was to discuss convergent mechanisms of immune regulation and T cell reprogramming in cancer and autoimmunity, to identify and dissect programs in immune cells that determine their tissue function and to define which capacities immune cells need to cure diseased tissues. With this goal about 150 scientists discussed over two days the recent developments in synthetic biology approaches and genetic engineering of immune cells ranging from sensor systems towards effector functions for the development of environment-smart immune cell therapies to treat cancer and autoimmune disorders.

## Improving CAR and TCR redirected T cell therapy of leukemia/lymphoma

Until now, more than 1000 patients were treated with anti-CD19 CAR T cells in the US alone; more than 270 trials are actively exploring CAR T cells in the treatment of hematologic and solid cancer around the world. Appreciating the success of CAR T cell therapy, Carl H. June (Center for Cellular Immunotherapies, University of Pennsylvania, Philadelphia, USA) reviewed in a keynote lecture the history of CAR T cells and highlighted the immune response of patients with metastatic colorectal cancer treated with first-generation CD3$\zeta$-chain CAR T cells targeting the tumor-associated glycoprotein-72 (TAG-72), one of the first human CAR T cell trials in the treatment of solid tumors performed in the 1990s. Targeting TAG-72 by CAR T cells seemed to be safe; in some patients there was a precipitous decline of TAG-72 serum levels indicating some anti-tumor response. However, CAR T cells showed limited persistence demanding the incorporation of a co-stimulatory domain and the use of a fully human CAR construct to mitigate anti-CAR immunogenicity. Long-term persistence is still a major issue for CAR T cell therapy; in lymphoma/leukemia trials CD27^+^ PD1^−^ CD8^+^ CAR T cells seem to be prognostic for long-term CAR T cell persistence and efficacy. Dr June also reported on an alternative way how to improve CAR T cell persistence. By in-depth analysis of a persistent CAR T cell clone in a patient his team identified that the CAR encoding sequence disrupted the TET-2 (Ten-Eleven-Translocation-2) gene that mediates DNA demethylation and is a master regulator in myelopoiesis and a tumor suppressor gene. Such CAR T cells exhibited a central memory phenotype and an epigenetic profile consistent with altered T cell differentiation. Experimental knock-down of the TET-2 gene improves CAR T cell function and persistence paving the way for a rational design of long-term persistent CAR T cells.

The level of targeted CD19 determines the efficacy of anti-CD19 CAR T cell therapy of B cell malignancies as pointed out by Michael C. Jensen (Seattle Children's Ben Towne Center for Childhood Cancer Research, Seattle Children's Research Institute, Seattle, WA, USA). The in-depth analysis of a phase I trial with a defined composition of CD19 CAR T cells revealed that the durability of remission correlated with engineered cell products containing increased frequencies of TNF-α secreting CD8^+^ CAR T cells. The therapeutic efficacy was moreover dependent on high CD19 levels at the time of infusion to trigger CAR T cell proliferation. T cells artificially expressing truncated CD19 stimulated anti-CD19 CAR T cells and improved their persistence and functionality in vivo. Dr Jensen suggested that re-stimulation by healthy B cells through a co-expressed CD19 CAR may enhance the efficacy of a CAR T cell attack against solid tumors. Beyond CD19 other targets are currently explored for the treatment of lymphoma/leukemia by CAR T cells. With this respect, Marcela V. Maus (Massachusetts General Hospital and Harvard Medical School, Boston, MA, USA) reported recent data on CAR T cells targeting CD37 that were active against lymphoma/leukemia of both B and T cell origin without evidence of significant T cell fratricide; a phase I trial is going to be initiated.

CAR T cell efficacy can also be enhanced by combinations with other immuno-oncological approaches. Carl H. June reported co-treatment with the Bruton tyrosine kinase (BTK) inhibitor ibrutinib along with anti-CD19 CAR T cells improves responses against mantle cell lymphoma and B cell chronic lymphocytic leukemia. In line with these data Marcela V. Maus demonstrated that ibrutinib increases the in vivo persistence and anti-tumor activity of CAR T cells in model systems. The strategy thereby combines therapies paving the way to a two-pronged assault in patients with lymphoma. Dr Maus further reported that CAR T cells can also be engineered to secrete a bispecific T cell engager (BiTE) antibody to promote recruitment and activation of bystander T cells, resulting in the clearance of tumors with heterogenous expression of the CAR targeted antigen.

CAR T cell engineering and ex vivo amplification also has a major impact on their performance and persistence in vivo. A number of different protocols in GMP-conform manufacturing of CAR T cells are currently applied resulting in potentially different CAR T cell products as reviewed by Ulrike Koehl (Fraunhofer Institute for Cell Therapy and Immunology, Leipzig, Germany). She highlighted that in contrast to CAR T cells, NK cells can be used from allogeneic donors as an “off-the-shelf product” thereby being alternative effector cells for redirected cancer therapy. CAR modified NK cells are currently explored in 10 early phase clinical trials worldwide for the treatment of lymphoma/leukemia. The necessity for NK cell stimulation and activation by cytokines to improve their proliferation and persistence led to the development of optimized CAR constructs for NK cells. Both, cord blood-derived and primary human NK cells engineered with a CD19 CAR and additionally with IL-15, to enhance the NK cell activity, as well as inducible caspase-9 (iCas9) as suicide mechanism showed extended persistence and improved anti-leukemia/lymphoma activity in an ongoing clinical phase I/IIa trial (principle investigator: K. Rezvani) at the MD Anderson Cancer Center, Houston, USA.

CAR T cell induced neurotoxicity is a major issue in the CD19 CAR T cell therapy of leukemia/lymphoma; the pathophysiology is so far not elucidated. In particular, the potential role of brain endothelial cells and the increased permeability of the blood brain barrier upon anti-CD19 CAR T cell treatment is not fully understood. Carl H. June pointed out that T cells engineered with the 4-1BB CAR break the blood brain barrier less frequently than CD28 CAR T cells, which may be responsible for the different frequency and severity of neurotoxicity. Moreover, in Dr June’s unpublished studies, brain pericytes were shown to have CD19 RNA transcripts suggesting that these cells may express CD19, although at low levels, and may thereby be potential targets of CD19 CAR T cells.

## CAR T cells on the road to target solid tumors

Targeting solid tumor lesions by CAR or TCR redirected T cells is still a major issue; various hurdles were identified and strategies developed during the last years. Apart from active immune repression, lack or low levels of targetable antigen is a common mechanism of cancer cells to become invisible to redirected T cells. To provide targetable antigen, Michael C. Jensen developed a technology to decorate tumor cells with a synthetic phospholipid that can be recognized by CAR modified T cells. Cancer cells with reduced membrane turnover like tumor cells preferentially retain this phospholipid as compared to healthy cells. Phospholipid-specific CAR T cells destroy such decorated cancers selectivity without the need of a targetable tumor-associated antigen. The strategy may contribute to make cancer lesions better visible to redirected T cells resulting in a more successful CAR T cell therapy in particular of solid cancer.

CAR T cells are more and more understood as living drugs redirected towards defined targets. Based on this concept CARs of the “fourth generation” were designed, also called TRUCKs or armored CARs, to deliver cytokines like IL-12 or IL-18 to the targeted tumor lesions as reviewed by **Hinrich Abken** (Regensburg Center for Interventional Immunology, Regensburg, Germany). In experimental models, inducible transgenic single-chain p40-p35 IL-12 locally deposited by CAR T cells upon CAR signaling attracted and activated innate cells eliminating antigen-negative cancer cells that are invisible to CAR T cells. The therapeutic effect is specific and locally restricted since tumor lesions without CAR targetable antigen were not attacked. In experimental systems inducible release of transgenic IL-18 converted effector T cells to T-bet^high^ FoxO1^low^ cells with improved cytolytic activities and prolonged persistence making the iIL-18 TRUCKs capable to eradicate advanced solid tumors that were not reduced by conventional CAR T cells. In a further development and to impact the immune environment of the targeted tumor lesion a “next generation” dual targeting CAR was introduced that is capable to target CD30 along with a tumor antigen. The CAR harbors an anti-CD30 scFv, that blocks the interaction with its ligand CD30L on T cells, and a tumor targeting scFv, both tandemly aligned in the same CAR binding domain. Blocking CD30 and targeting a cancer antigen by the same CAR improves the T cell response against CD30-negative solid tumors. This is a first example of a strategy to improve redirected cell therapy by modulating the T cell response itself by a multi-valent CAR.

## Engineering the most suitable T cell capacities for an anti-tumor attack

Leukemia/lymphoma is currently also treated by adoptive transfer of TCR engineered T cells as reviewed by **Dolores J. Schendel** (Medigene Immunotherapies GmbH, Medigene AG, Planegg, Germany). She reported on an ongoing clinical trial using PRAME-specific TCR T cells for the treatment of AML, MDS and multiple myeloma. High-affinity TCRs are key for the success of TCR modified T cells; tremendous efforts to identify suitable TCRs are currently undertaken in different laboratories with different strategies. Dr Schendel discussed how naturally occurring high-affinity TCRs can be efficiently identified in a co-culture system using dendritic cells co-transfected with RNA encoding an allogeneic MHC molecule and a tumor-associated antigen. New technological advances allow for automated and sensitive screening of thousands of such co-cultures to facilitate the selection of TCRs with optimal affinities. Like CAR T cells, TCR modified T cells benefit from additionally providing appropriate costimuli to enhance and prolong activation. Based on this concept Dr Schendel reported on the engineering with a PD-1/4-1BB switch receptor that binds through PD-1 and signals through 4-1BB costimulation to improve repetitive killing capacities and persistence of such engineered T cells.

At the University College London T cells engineered with a WT-1 specific TCR are currently clinically explored for the treatment of leukemia patients as reviewed by **Hans J. Stauss** (Institute of Immunity and Transplantation, Division of Infection and Immunity, University College London, London, UK). To improve the anti-tumor capacity in a timely and locally restricted fashion he presented two strategies. In an experimental system he demonstrated how a tetracycline-inducible system can be employed for safe and effective delivery of IL-12 into the tumor microenvironment by adoptively transferred T cells. Upon tetracycline administration, IL-12 released by the engineered T cells in the tumor tissue boosts the anti-tumor response. The second strategy is aiming at modulating mTOR signaling in transferred T cells by targeting RHEB and PRAS40. Increasing mTOR signaling by enforced expression of RHEB promotes aerobic glycolysis and augments the expansion and tumor infiltration by effector cells, reducing the emergence of immuno-edited escape variants. Conversely, ectopic expression of PRAS40 temper mTOR signaling favoring the generation of long-lived memory T cells and recall immunity. In line with that **Luca Gattinoni** (Center for Cancer Research, NCI, NIH, Bethesda, MD, USA) discussed how manipulation of the microRNA-epigenetic regulatory circuitry could be an effective strategy to enhance T cell anti-tumor function. He showed that overexpression of miRNA-155, an essential miRNA for CD8^+^ T cell anti-viral and anti-tumor immunity, promotes cancer regression in the absence of lymphodepletion and exogenous cytokine administration. miRNA-155 augments T cell expansion, survival and sustains T cell production of pro-inflammatory cytokines. Mechanistically, he demonstrated that miRNA-155 enhances T cell responsiveness to homeostatic cytokines by repressing the translation of several inhibitors of cytokine signaling, including SOCS1, PTPN2 and SHIP-1. Moreover, inhibition of SHIP1 indirectly promotes PRC2 activity through the induction of the PRC2-associated factor PHF19 via AKT signaling, resulting in the epigenetic silencing of programs of senescence and exhaustion. Finally, PHF19 could be directly harnessed to epigenetically reprogram CD8^+^ T cell fate and to potentiate T cell-based immunotherapy.

A strategy to improve the visibility of cancer cells to redirected T cells was presented by **Reno Debets** (Department of Medical Oncology, Erasmus MC Cancer Institute, Rotterdam, The Netherlands). In experimental systems the combination of TCR T cells and epigenetic drug-treatment of tumor cells with the epigenetic drugs azacytidine and valproate improved the antigenicity of cancer cells and consequently the T cell response against tumors. A clinical trial is currently developed for the treatment of metastasized melanoma and head-and-neck carcinoma using MAGE-C2/HLA-A2 specific TCR T cells and azacytidine and valproate co-application. Both, the target antigen MAGE-C2, and the patient-derived TCR16, are currently passing stringent safety assessments to limit risks of on- and off-target toxicities.

As an evolutionay conserved and highly efficacious mechanism against pathogens, alloreactive T cells are exploited in the adoptive cell therapy of cancer. Allorepertoire derived T cells were used to identify TCRs recognizing cancer cell derived peptides in HLA-A2. T cells were engineered with such allorestricted TCR specific for chondromodulin-I/HLA-A*02:01 to target metastatic Ewing sarcoma cells as reported by **Stefan Burdach** (Department of Pediatrics and Children's Cancer Research Center, Kinderklinik München Schwabing, Technische Universität München, Munich, Germany). Engineered allorestricted T cells homed to the affected bone marrow (in one out of three patients) resulting in partial disease regression without inducing graft-versus-host disease. Chondromodulin-I is an ideal target as it plays a critical role in Ewing sarcoma pathogenesis by being upregulated by the Ewing sarcoma fusion oncogene EWS-FLI1 and by maintaining an undifferentiated invasive phenotype and metastatic spread of the sarcoma cells. Dr Burdach further reported on transcriptome analyses of pediatric refractory sarcomas (Ewing sarcoma, osteosarcoma, soft tissue sarcoma) aiming at identifying targetable overexpressed genes and deregulated pathways. First data exemplarily demonstrate that such individualized tumor profiling can provide a rationale in an individualized combination of targeted therapies and thereby guide the treatment options. First treated patients with refractory sarcoma seem to benefit from such designed therapies; further clinical evaluations are ongoing.

## Treg cells to control auto-immune diseases

In a keynote lecture, **Shimon Sakaguchi** (WPI Immunology Frontier Research Center, Osaka University, Osaka, Japan) reviewed the diversity and function of human Treg cells. In cancer patients Treg cell depletion may be beneficial; in addition to current Treg cell depletion strategies Dr Sakaguchi suggested depleting antibodies against CCR4 as an effective way to eliminate effector Treg cells. On the other hand inducing Treg cells may help to control auto-immune diseases. In this context Dr Sakaguchi reported about the identification of a small molecule that allowed the induction of FOXP3 in conventional CD4^+^ and CD8^+^ T cells in the absence of TGFβ. The compound was able to convert already differentiated effector T cells into FOXP3 expressing Treg cells. In different experimental in vivo model systems, e.g., of type-1 diabetes and contact hypersensitivity, this small molecule showed a beneficial effect. Mechanistically, the compound inhibited CDK8/19, which inhibited phosphorylation of STAT5. In turn, phosphorylation of STAT5 tyrosine residue induced FOXP3 expression by interacting with the CNS-0 region of the FOXP3 promoter. The compound is of great interest for the conversion of effector T cells into FOXP3 expressing Treg cells as a potential therapeutic option for a variety of auto-immune and chronic inflammatory diseases in different tissues. In this context **Markus Feuerer** (Regensburg Center for Interventional Immunology, Regensburg, Germany) reported on the specialization of Treg cells in different tissues. Based on whole genome DNA-methylation studies he described about 11,000 regions that were differentially methylated when tissue Treg cell populations and lymphoid T cells were pairwise compared. Similarities in the epigenetic landscape led to the identification of a common tissue Treg cell population present in virtually all organs. This common tissue Treg cell population was characterized by loss of DNA methylation that included many gene sites associated with the Th2 subset of helper T cells, such GATA3 and IRF4, and were characterized by the expression of the IL-33 receptor ST2. These ST2^+^ tissue Treg cells produced tissue-regenerative factors such as amphiregulin. Using a novel reporter mouse line, single-cell RNA-sequencing and ATAC-sequencing, he introduced precursor stages of this non-lymphoid tissue Treg population in spleen and lymph nodes.

Tissue Treg cells with tissue-regenerative potential are of interest for therapeutic interventions in situations of tissue destruction and chronic disturbance of tissue homeostasis. This, however, requires tissue directed targeting of Treg cells towards pre-defined organs. Research in this direction was presented by **Megan K. Levings** (BC Children's Hospital Research Institute, University of British Columbia, Vancouver, BC, Canada) reporting data on the modification of host-derived Treg cells with CARs directed against HLA class I mismatches for the induction of tolerance in HLA-mismatched solid organ transplantation. Using xenogenic skin graft models she showed that CAR-modified Treg cells are superior as compared to unmodified polyclonal Treg cells in HLA-transgenic skin transplantation models and human skin xenografts on NSG mice. CAR-modified Treg cells were shown to infiltrate and survive in the graft and to inhibit keratinocyte proliferation. Furthermore, such CAR Treg cells accumulated in newly generated lymph nodes draining the transplanted tissue where they seem to inhibit APC function and thereby the perpetuation of the allo-response.

The role of thymus-derived donor Treg cells in allogeneic bone marrow transplantation (BMT) was discussed by **Matthias Edinger** (Regensburg Center for Interventional Immunology and Clinic III Internal Medicine, University Hospital Regensburg, Regensburg, Germany) who showed that Treg cells contained in the original bone marrow graft are pivotal for the long-term tolerance after haploidentical BMT in murine models. He demonstrated that they engraft long-term and actively suppress allo-responses of co-transplanted conventional donor T cells. In vitro expanded donor Treg cells efficiently ameliorate ongoing acute GvHD after MHC-mismatched BMT and rescue two thirds of mice from otherwise lethal acute GvHD. These findings are now tested in two clinical trials exploring the efficacy of in vitro expanded donor Treg cells for the treatment of patients with acute and chronic GvHD. Preliminary data from those clinical trials seem to confirm the therapeutic effects of donor Treg cell transfers.

The T cell populations including Treg cells infiltrating tumor lesions are extensively heterogenous, however, Tr1 CD4^+^ T cells are abundantly present in tumors. This became obvious by extensive analyses reported by Massimiliano Pagani (Istituto Nazionale Genetica Molecolare, Milano, Italy). Using single cell RNA sequencing technology he identified and characterized T helper-1 (Th1), Th17, and Treg cell populations infiltrating colorectal and non-small-cell lung cancers. He suggested a functional role for Tr1 cells in suppressing anti-tumor immune responses in situ. Apart from Treg cells, interstitial macrophages reside within the tissue parenchyma. Florent Ginhoux (Singapore Immunology Network, Singapore, Singapore) introduced two independent monocyte-derived interstitial macrophage subpopulations which are conserved across different tissues. One population is mostly located adjacent to nerves, whereas the other resides close to blood vessels, each performing specific non-redundant functions important for the integrity of the tissue. Furthermore, he discussed recent data by his laboratory and work from other groups describing the origin of plasmacytoid dendritic cells (pDCs). pDCs develop from lymphoid progenitors and, thereby, are distinct from the myeloid lineage, implicating that pDCs are not a subtype of DCs and are rather an innate lymphoid cell (ILC) type. Consequently, pDCs should be renamed and integrated in the innate lymphoid cell spectrum, e.g., renamed as ILC4 or ILC-IFNα producer cells. The new origin and marker definition might also help to resolve controversial findings about pDC functions which could be explained by contamination of the pDC sort fraction with true DC precursor cells leading to a heterogeneous cell mix with APC potential.

## Applied bioinformatics meets synthetic immunology

Advanced combined bioinformatic and genetic approaches are currently applied to determine individual and immunogenic mutations in even small tumor cell subclones. **Benedikt Brors** (Division of Applied Bioinformatics, German Cancer Research Center DKFZ, Heidelberg, Germany) outlined novel algorithms established at the National Center for Tumor diseases in Heidelberg. Such approaches are essential to identify individual treatment opportunities for both molecular targeted therapies and/or mutation-specific cancer immunotherapies. First data suggest a potential superiority of personalized treatment over conventional therapies.

To engineer the ideal designer cell for adoptive therapy different approaches of genome engineering with CRISPR/Cas9 technology are currently explored. **Tudor Fulga** (Weatherall Institute of Molecular Medicine, Radcliffe Department of Medicine, University of Oxford, Oxford, UK) reviewed in a keynote lecture different approaches of genome engineering with CRISPR/Cas9 technology and artificial cell reprogramming by post-transcriptional regulation of gene expression. Strategies are currently established to encode specific transcriptional programs by generating inducible Cas9 transcription factors through engineering of CRISPR sgRNAs. Furthermore, he described the engineering of a new class of chimeric, catalytically inactivated Cas9 (nuclease dead Cas9, dCas9) synthetic receptors that can sense various native ligands. He introduced the concept of miRNA silencing-mediated fine-tuners called miSFITs which enable tightly controlled expression levels of target genes. As an example Dr Fulga demonstrated to tune the T cell inhibitory PD-1 and to explore how antigen expression influences T cell activation and tumor growth. In principle, all these novel technologies can be applied to modulate and enhance CAR T cell therapy, however, will still acquire some T cell specific adaptations.

## Conclusions

Taken together the Symposium clearly showed that adoptive cell therapy with engineered T cells against solid organ tumors is the next frontier in cancer therapy. If successful, it will dramatically change the way how cancer patients are treated. As living drugs, CAR T cells have the potential for long-term persistence and for delivering immune active products. So-called TRUCKs, T cells redirected for unrestricted cytokine mediated killing, or armored CAR T cells may be used to modulate the targeted tissue by depositing cytokines or other proteins. The here described advances in T cell engineering, genetic editing, the selection of optimal lymphocyte subsets, and cell manufacturing have the potential to broaden immune cell–based therapies and foster new applications far beyond oncology, in particular for the treatment of infectious diseases, organ transplant rejection, and autoimmunity.

## Abbreviations

AKT: Protein kinase B
AML: Acute myeloid leukemia
APC: Antigen presenting cell
ATTAC: Assay for transposase accessible chromatin
BiTE: Bispecific T cell engager
BTK: Bruton tyrosine kinase
CAR: Chimeric antigen receptor
CCR4: C–C motif chemokine receptor-4
CDK: Cyclin dependent kinase
CRISPR: Clustered regularly interspaced short palindromic repeats
DC: Dendritic cell
EWS: Ewing sarcoma
FLI1: Friend leukemia integration-1
FoxO1: Forkhead box protein-O1
FoxP3: Forkhead box protein-P3
GMP: Good manufacturing practice
GvHD: Graft versus host disease
iCAS9: Inducible caspase-9
IRF4: Interferon regulatory factor-4
MAGE-C2: Melanoma associated antigen-C2
MDS: Myelodysplastic syndrome
miRNA: MicroRNA
miSFITs: MicroRNA silencing-mediated fine-tuners
mTOR: Mechanistic target for rapamycin
NSG: Non-obese diabetic severe combined immunodeficiency gamma
PD-1: Programmed cell death-1
pDC: Plasmacytoid dendritic cell
PHF19: PHD finger protein-19
PRAME: Preferentially expressed antigen in melanoma
PRAS40: Proline-rich AKT substrate of 40 kD
PRC2: Polycomb repressive complex-2
PTPN2: Protein tyrosine phosphatase non-receptor type-2
RCI: Regensburg Center for Interventional Immunology
RHEB: RAs homolog enriched in brain
scFv: Single chain fragment of variable region
sgRNA: Single guide RNA
SHIP-1: Src homology phosphoinositide phosphatase-1
SOCS1: Suppressor of cytokine signaling-1
STAT5: Signal transducer and activator of transcription-5
TAG-72: Tumor-associated glycoprotein-72
Tbet: T cell specific T-box transcription factor
TCR: T cell receptor
TET-2: Ten-eleven-translocation-2
TGF-β: Transforming growth factor-β
Th: T helper
TRUCK: T cell redirected for unrestricted cytokine-mediated killing
WT-1: Wilms tumor-1

